# Quantifying the Acoustic Startle Response in Mice Using Standard Digital Video

**DOI:** 10.3389/fnbeh.2020.00083

**Published:** 2020-06-03

**Authors:** Madeline M. Pantoni, Gerald M. Herrera, Kaitlin R. Van Alstyne, Stephan G. Anagnostaras

**Affiliations:** ^1^Molecular Cognition Laboratory, Department of Psychology, University of California, San Diego, La Jolla, CA, United States; ^2^Med-Associates Inc., Catamount Research & Development Inc., St. Albans, VT, United States; ^3^Department of Pharmacology, University of Vermont, Burlington, VT, United States; ^4^Program in Neurosciences, University of California, San Diego, La Jolla, CA, United States

**Keywords:** startle, prepulse inhibition, video, methods, rodent models, phenotyping, fear conditioning, neuropsychiatric disorders

## Abstract

The startle response is an unconditional reflex, characterized by the rapid contraction of facial and skeletal muscles, to a sudden and intense startling stimulus. It is an especially useful tool in translational research for its consistency across species, simple neural circuitry, and sensitivity to a variety of experimental manipulations. The rodent acoustic startle response is commonly used to study fundamental properties of the central nervous system, including habituation, sensitization, classical conditioning, fear and anxiety, sensorimotor gating, and drug effects. The rodent startle response is typically assessed in stabilimeter chambers, and while these systems are excellent at measuring startle, they are designed only for this sole purpose. In the present study, we used the VideoFreeze system—a widely used tool for studying Pavlovian fear conditioning—to assess the acoustic startle response in freely moving mice. We validated the use of this system to quantify startle response amplitude and prepulse inhibition of startle. This is the first demonstration to date of using standard video in the automated assessment of the acoustic startle response in rodents. We believe that researchers already using the VideoFreeze system will benefit from the additional ability to assess startle without the purchase of new equipment.

## Introduction

The startle response is an unconditional reflex, characterized by the rapid contraction of facial and skeletal muscles, to a sudden and intense startling stimulus, such as a noise burst, air puff, or light flash (Landis and Hunt, 1939; Koch and Schnitzler, 1997; Berg and Balaban, 1999; Swerdlow et al., 1999). It is an especially useful tool in translational research for its consistency across species (Landis and Hunt, 1939; Bullock, 1984; Davis, 1984; Swerdlow et al., 1999), simple neural circuitry (Davis et al., 1982; Lingenhohl and Friauf, 1994; Yeomans and Frankland, 1995; Koch and Schnitzler, 1997), and sensitivity to a variety of experimental manipulations (Koch and Schnitzler, 1997; Koch, 1999; Fendt and Koch, 2013). The rodent acoustic startle response is commonly used to study fundamental properties of the central nervous system, including habituation, sensitization, classical conditioning, fear and anxiety, sensorimotor gating, and drug effects (Groves and Thompson, 1970; Davis, 1980, 1986, 2006; Davis et al., 1982, 1993; Swerdlow et al., 1992; Pilz and Schnitzler, 1996; Koch, 1999). One important phenomenon that is used to model sensorimotor gating is prepulse inhibition (PPI), the suppression of the startle response when a weak prestimulus precedes the strong startling stimulus (Graham, 1975; Swerdlow et al., 2000; Li et al., 2009). Deficits in sensorimotor gating are important features of many neuropsychiatric disorders (e.g., schizophrenia, obsessive compulsive disorder, Huntington's disease, Tourette syndrome) (see review by Kohl et al., 2013), and thus PPI of the rodent acoustic startle response has become a leading tool for studying the pathophysiology, pharmacology, and genetics of these disorders (Swerdlow and Geyer, 1998; Swerdlow et al., 2000, 2016; Geyer et al., 2001, 2002; Powell et al., 2011; Fendt and Koch, 2013).

Assessing the startle response in rodents can be challenging given its extremely brief duration. The latency of the rodent acoustic startle response is estimated to be between 5 and 12 ms among different muscle groups (e.g., neck, hindlimb) (Ison et al., 1973; Willott et al., 1979; Davis et al., 1982; Cassella et al., 1986; Parham and Willott, 1988; Lingenhohl and Friauf, 1994; Yeomans and Frankland, 1995; Pilz and Schnitzler, 1996; Koch and Schnitzler, 1997; Carlson and Willott, 1998). Because of this challenge, the rodent startle response is typically assessed in small stabilimeter chambers that constrain animal movement (Geyer and Swerdlow, 1998; Geyer and Dulawa, 2003). This testing process can be stressful and unpleasant for animals and requires extensive habituation and calming procedures (Geyer and Swerdlow, 1998). Moreover, this chamber is designed only to measure this single behavior. Thus, the ability to measure rodent startle intensity using alternative methods such as standard video in a Skinner-type conditioning chamber could be exceptionally valuable.

In the present study, we validate the use of the VideoFreeze system (Med-Associates Inc., Georgia, VT, USA) to assess the acoustic startle response and detect PPI of this response in freely moving mice. This system was designed for the automated assessment of freezing behavior and locomotor activity using digital video (see Anagnostaras et al., 2010). Animal movement within the digital video stream is quantified using a motion index, which is generated using a proprietary motion analysis algorithm that compares successive video frames while controlling for baseline video noise on a pixel-by-pixel basis. VideoFreeze is quite sensitive in scoring rodent movements of any kind, including ultra-fine movements such as respiration. VideoFreeze samples video at 30 Hz, and at face value, it may seem that the acoustic startle response is too fast to capture using standard digital video. However, the VideoFreeze system time locks stimulus presentation with the timing of video frame acquisition, and the exposure time per frame is relatively long. Thus, it is plausible that the 30 Hz video stream would capture frames just before, during, and immediately after the startle response, and then could be used to score startle intensity. Indeed, we found that the VideoFreeze system accurately measured the startle response and PPI of this response in mice. Although the traditional floor deflection potentiometer startle systems are excellent at measuring startle responses, they are also complex, specialized only for startle, expensive, and take up lab space. We suggest this advancement could be useful for labs that already own VideoFreeze systems and may want to evaluate startle.

## Materials and Methods

### Subjects

16 (8 males, 8 females) hybrid C57BL/6Jx129S1/SvImJ (Jackson Laboratory, West Sacramento, CA, USA) mice were used. Mice were weaned at 3 weeks of age and group-housed (2–5 mice per cage) with unrestricted access to food and water. The animal colony was kept on a 14:10-h light/dark schedule and all testing occurred during the light phase. Mice were at least 10 weeks old and handled for 3 days (1 min/day) prior to testing. All animal care and experimental procedures were approved by the UCSD IACUC and in compliance with the NRC 8^th^ *Guide for the Care and Use of Laboratory Animals*.

### VideoFreeze System

The VideoFreeze system (Med-Associates Inc., Georgia, VT, USA; see Anagnostaras et al., 2010) was used to assess acoustic startle. For all experiments, four mice were tested concurrently in individual chambers (32 × 25 × 25 cm), which consisted of stainless-steel side walls and rod floors, white acrylic back walls, and clear polycarbonate front and top walls. Testing chambers were illuminated with white and near-infrared light and were cleaned with 7% isopropyl alcohol. Each chamber was encased in a sound-attenuated box, and background noise (65 to 70 dB) was produced by internal ventilation fans. A broad band white noise signal generated by VideoFreeze was rerouted through a consumer amplifier (80W RMS per speaker; Denon DRA-395) and sent to consumer speakers (2.75-inch woofer, 0.5-inch tweeter; Yamaha NS-AP1400S) placed inside each chamber. Testing sessions were video recorded at a rate of 30 Hz by a standard digital camera mounted in front of each chamber and connected to a Windows computer running the VideoFreeze software (Med Associates Video Freeze Software, RRID:SCR_014574, SOF-843). VideoFreeze used this video stream to quantify animal movement via a motion index (see Motion Scoring section below).

### Input/Output Function

A protocol adapted from Valsamis and Schmid (2011) was used to generate an input/output (i/o) function for our hybrid mouse colony, which represents the relationship between acoustic stimulus intensity and startle response amplitude. Mice were habituated to both the testing chambers and the acoustic stimuli twice prior to i/o function testing. The acoustic stimuli were 200 ms white noise bursts with 0 ms rise times that varied in decibel intensities. Testing began with a 4-min baseline period, followed by the presentation of one noise burst every 20 s, which started at 75 dB and increased between each presentation by 5 dB until reaching 120 dB. The 75 dB noise burst was presented four times and all other noise bursts were presented only once.

### High-Speed Video

A separate observation of the startle response was conducted using a high-speed imaging system to observe the response with greater temporal resolution. A MotionBLITZ EoSens mini camera (Mikrotron, Munich, Germany) was used to record video at 1,000 Hz. Video acquisition was triggered by an output from VideoFreeze using a 28-volt to TTL converter (SG-231, Med Associates) so that the high-speed video could be correlated with the timing of startle stimulus presentation. The VideoFreeze system was running simultaneously so that the videos and data from the high-speed imaging system and the VideoFreeze system could be compared. The primary purpose of this was to ensure the startle response we were recording accords well with that recorded in standard startle chambers.

### Prepulse Inhibition

**Figure 1:**
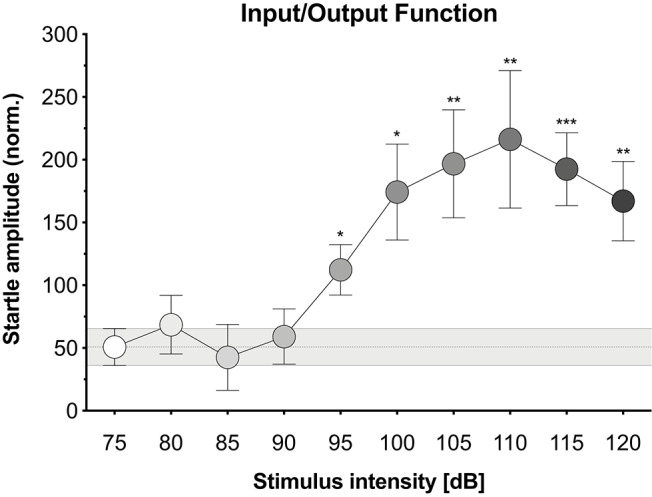
Input/output function. The relationship between acoustic stimulus intensity and startle response amplitude for our hybrid mice. Acoustic stimuli were white noise bursts (200 ms with 0 ms rise time) of increasing intensities (four trials at 75 dB then single trials at 80–120 dB). Normalized startle amplitude was significantly enhanced at the 95, 100, 105, 110, 115, and 120 dB noise bursts relative to the 75 dB noise burst. Each point represents the mean ± 1 standard error. The gray bar indicates standard error range for the comparison 75 dB noise burst. Data points with asterisk identify significant comparisons against the 75 dB noise burst using Fisher's LSD (*P < 0.05, **P < 0.01, and ***P < 0.001).

A protocol adapted from Valsamis and Schmid (2011) was used to assess prepulse inhibition (PPI) of the acoustic startle response. Based on the i/o function (see Results section, Figure 1), the 105 dB noise burst produced significant startle and the 75 and 85 dB noise bursts produced little to no startle. Accordingly, 105 dB white noise bursts (200 ms duration, 0 ms rise time) were used as pulse stimuli and 75 or 85 dB white noise bursts (4 ms duration, 0 ms rise time) were used as prepulse stimuli.

PPI testing began with a 5-min baseline, followed by a habituation phase and then a PPI phase. The habituation phase consisted of the presentation of 30 pulses, each 20 s apart. The PPI phase consisted of 50 trials—pulse-only trials (10) and prepulse/pulse trials (40)—each 20 s apart. In the prepulse/pulse trials, the prepulse was presented prior to the pulse at an inter-stimulus interval (ISI) of 50 or 100 ms. The 50 trials were pseudorandomized into five conditions: (1) No prepulse (pulse-only), (2) 75 dB prepulse and 50 ms ISI, (3) 85 dB prepulse and 50 ms ISI, (4) 75 dB prepulse and 100 ms ISI, or (5) 85 dB prepulse and 100 ms ISI.

An additional prepulses-only experiment was conducted to determine the effect of the prepulses alone on startle. This experiment began with a 5-min baseline, followed by 20 prepulse-only trials, each 20 s apart, that alternated between 75 and 85 dB.

### Motion Scoring

VideoFreeze uses a proprietary motion analysis algorithm (see (Anagnostaras et al., 2010) for full description) to calculate a motion index (in arbitrary units [au]) for each frame of video, which measures the number of changed pixels between successive video frames while ignoring pixel changes caused by video noise (primarily jitter and compression artifacts). A reference video sample is taken before an animal is placed in the conditioning chamber in order to establish the amount of baseline noise inherent to the video signal. This approach determines the number of pixels in which the intensity value is changing from frame to frame under baseline (no animal present) conditions. Once the animal is placed in the chamber, the number of pixels in which the intensity value is changing from frame to frame is compared against the baseline noise reference. The motion index represents the number of pixels that are changing from frame to frame above the baseline noise level. Consequently, a frame in which a large movement occurs results in a high motion index, and because the camera accumulates exposure across each shutter interval, this movement appears as blur in the video still image.

The maximum motion index value within a specified time frame was used to score startle amplitude, as this measure captures rapid yet significant alterations in movement that occur in response to the onset of a noise burst. The maximum motion index during the time frame of interest (i.e., during the noise burst) was normalized to the maximum motion index during a baseline period (i.e., immediately prior to the noise burst). Despite the brevity of the mouse startle response (see Introduction), it is advised to measure whole-body startle over a relatively long interval (e.g., 100 to 200 ms) after stimulus onset (Cassella et al., 1986). For the i/o experiment, normalized startle amplitude was calculated as the maximum motion index during the 200 ms *after* the onset of the noise burst minus the maximum motion index during the 200 ms *before* the onset of the noise burst. For the PPI experiment, normalized startle amplitude was calculated as the maximum motion index during the 200 ms *after* the onset of the pulse minus the maximum motion index during the 200 ms *before* the onset of the prepulse (300 to 100 ms before the onset of the pulse). For the prepulses-only experiment, normalized startle amplitude was calculated as the maximum motion index during the 200 ms *after* the onset of the prepulse minus the maximum motion index during the 200 ms *before* the onset of the prepulse.

A motion index was also calculated for each frame of the high-speed video stream. Here, each video frame (a region of interest containing the mouse) was compared to the background video (same sized region, but no mouse) on a pixel-by-pixel basis and expressed as an overall ratio, such that a motion index of 1 represents animal motion that is similar to the background level of video noise. The pseudocoloring of the video frame pixels in **Figure 3** is scaled according to how much each pixel varies from the background video signal, with brighter colors (i.e., yellow) indicating more animal motion in that region.

### Statistical Analyses

Data were analyzed using repeated measures univariate analyses of variance (ANOVAs) to identify overall group differences. *Post-hoc* comparisons were performed following significant ANOVAs using Fisher's Least Significant Difference (LSD) tests against a control condition (75 dB noise burst in the i/o experiment; pulse-only, pulse/prepulse at the same intensity, and prepulse-only at the same intensity in the PPI experiment). The level of significance was set at *p* ≤ 0.05 for all analyses.

## Results

We first explored the potential to elicit and measure the acoustic startle response using the Video Freeze system. Mice were presented with white noise burst stimuli of increasing intensities and movement was quantified via motion index scores derived from the video signal. Figure 1 displays the i/o function for our hybrid mice, which established the average normalized startle amplitude in response to acoustic stimuli of increasing intensities (see Supplementary Data 1 for corresponding data sheet and Supplementary Figure 1 for scatterplot of individual animal data). Normalized startle amplitude differed significantly across stimulus intensities [*F*_(3.894, 58.41)_ = 5.325, *p* = 0.001]. The 95, 100, 105, 110, 115, and 120 dB noise bursts led to significantly higher normalized startle amplitudes than the 75 dB noise burst (*p*-values ≤ 0.012). The 80, 85, and 90 dB noise bursts had no effect on normalized startle amplitude relative to the 75 dB noise burst (*p*-values ≥ 0.524). To address concerns regarding the robustness of these measures in freely moving mice, we analyzed the effect of animal orientation on startle measurements. Animals were grouped by whether they were oriented forward, backward, or sideways during the 105, 110, and 115 dB noise bursts. Normalized startle amplitude did not significantly differ between animal orientations at all three stimulus intensities [see Supplementary Figure 2; 105 dB, *F*_(2, 13)_ = 0.079, *p* = 0.925; 110 dB, *F*_(2, 13)_ = 0.2, *p* = 0.821; 115 dB, *F*_(2, 13)_ = 0.976, *p* = 0.403].

**Figure 2:**
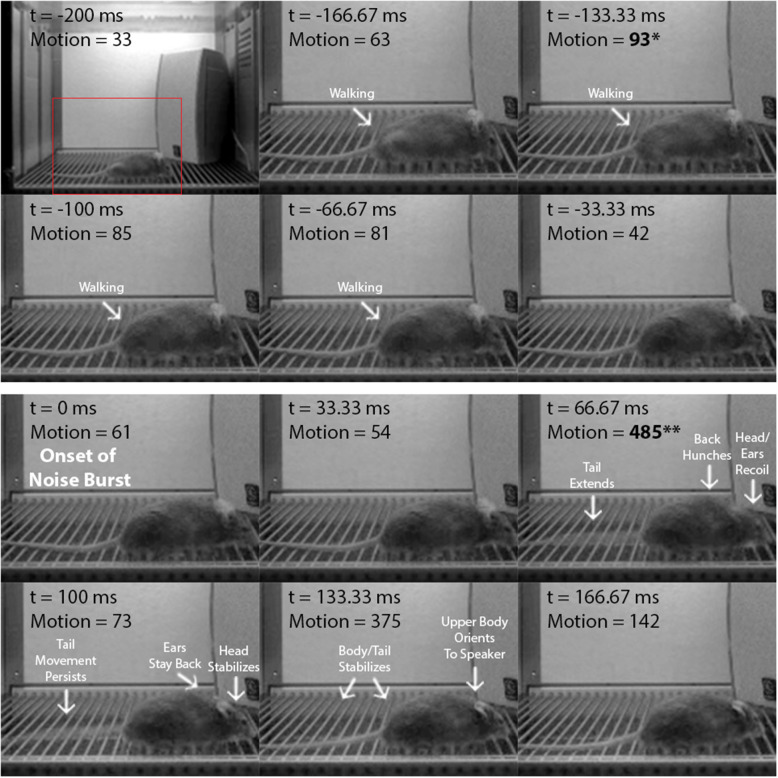
The startle response as captured by VideoFreeze. Frame-by-frame video still images showing a mouse from 200 ms before to 200 ms after the onset of the 105 dB, 200 ms white noise burst in the i/o experiment. Digital video was recorded at 30 Hz, so each frame represents 33.33 ms of video. Time (t) is relative to the onset of the noise burst (*t* = 0 ms). Motion represents the motion index score calculated by the VideoFreeze system. Top six frames include the 200 ms before the onset of the noise burst (maximum motion index = 93 au, indicated by asterisk) and bottom six frames include the 200 ms after the onset of the noise burst (maximum motion index = 485 au, indicated by double-asterisk). For this example, normalized startle amplitude (maximum motion index after minus before onset) = 392 au. Before the noise burst, baseline activity was captured by motion indexes of ≤ 93 au. At *t* = 66.67 ms, there was a large spike in the motion index to 485 au, and the startle response was observed and characterized by significant head, ear, back, and tail movements (body parts in motion appear blurry in image). At *t* = 100 ms, the motion index decreased to 73 au, as movement was observed in the tail only. The startle response concluded by *t* = 133.33 ms.

To confirm that the startle amplitude increases produced by the higher-intensity noise bursts in the i/o experiment accurately reflect the mouse startle response, we analyzed startle video recordings from: (1) VideoFreeze (i/o experiment), and (2) a high-speed camera (a separate experiment). Figure 2 is a frame-by-frame exhibition of a mouse startle response to a 105 dB, 200 ms noise burst, as recorded by VideoFreeze at 30 Hz during i/o testing (see Supplementary Movie 1). Each frame represents 33.33 ms of standard digital video and the VideoFreeze motion index for each frame is indicated. The 200 ms *before* (top six frames) and the 200 ms *after* (bottom six frames) the onset of the noise burst (*t* = 0 ms) are shown. Before the noise burst, baseline activity (i.e., walking) was captured by motion indexes of ≤ 93 au. The startle response was observed at *t* = 66.67 ms after the onset of the noise burst and was characterized by a rapid recoil of the head and ears, hunching of the back, and extension of the tail. Because the camera accumulates exposure across each shutter interval, this appears as a blur which is scored as a large movement by the VideoFreeze algorithm. Accordingly, at this same time point (*t* = 66.67 ms), there was a large spike in the motion index to 485 au. Nearly all of the startle response was captured within this 1 video frame except for some tail movement that was observed at *t* = 100 ms. In all, the startle response was clearly reflected by a large increase in the maximum motion index (485 au) relative to baseline (93 au), resulting in a normalized startle amplitude of 392 au.

**Figure 3:**
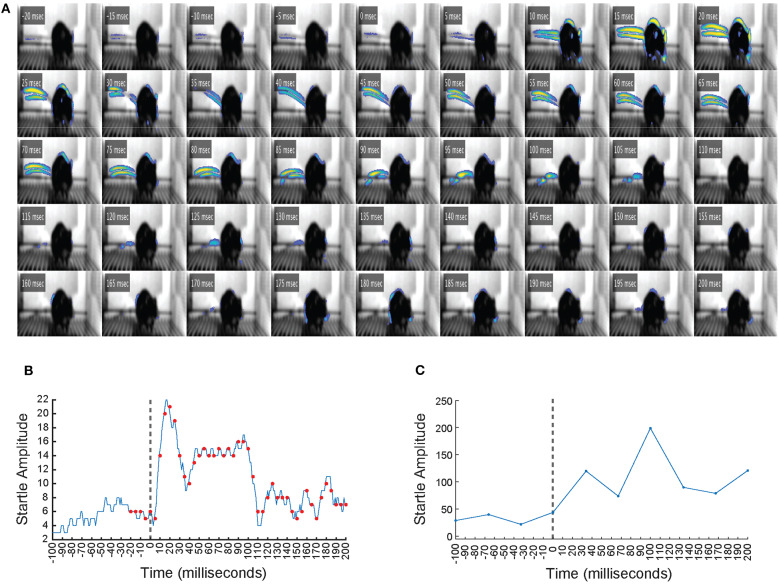
The startle response as captured by a high-speed imaging system. **(A)** Frame-by-frame video still images showing a mouse from 20 ms before to 200 ms after the onset of a 105 dB, 200 ms white noise burst. Digital video was recorded at 1,000 Hz, so each frame represents 1 ms of video. Time (t) indicated is relative to the onset of the noise burst (*t* = 0 ms). Animal motion is represented by pseudocoloring of the pixels, such that brighter colors indicate more movement in that region. The startle response was observed from *t* = 5 ms to *t* = 105 ms and was characterized by significant nose, ear, back, and tail movements that progressed from rostral to caudal. **(B)** The motion index calculated from the high-speed video of every 1 ms from 100 ms before to 200 ms after the onset of the noise burst. Vertical dashed line indicates the onset of the white noise burst at *t* = 0 ms. Red dots represent the time points of the video frames displayed in **(A)**. Startle amplitude showed a large increase between *t* = 0 ms and *t* = 200 ms. **(C)** The motion index calculated by VideoFreeze of every 33.33 ms from 100 ms before to 200 ms after the onset of the noise burst. Vertical dashed line indicates the onset of the white noise burst at *t* = 0 ms. Startle amplitude showed a large increase between *t* = 0 ms and *t* = 200 ms.

In a separate experiment, a high-speed camera that samples video at 1,000 Hz was used alongside VideoFreeze to observe the startle response with greater temporal resolution. Figure 3A is a frame-by-frame exhibition of a mouse startle response to a 105 dB, 200 ms noise burst, as recorded by the high-speed imaging system (see Supplementary Movie 2). Each frame represents 1 ms of digital video, and every fifth frame from 20 ms before to 200 ms after the onset of the noise burst (*t* = 0 ms) is shown. Animal motion is represented by pseudocoloring of the pixels, which was scaled according to how much the pixels varied from the background video signal, such that brighter colors indicate more movement in that region. Before the noise burst, very little movement was observed. The startle response was observed from *t* = 5 ms to *t* = 105 ms and was characterized by the same nose, ear, back, and tail movements observed in Figure 2, which progressed from rostral to caudal. Figure 3B presents the motion index calculated from the high-speed video of every 1 ms from 100 ms before to 200 ms after the onset of the noise burst. Figure 3C presents the motion index calculated by VideoFreeze of every 33.33 ms from 100 ms before to 200 ms after the onset of the noise burst. Both the high-speed (Figure 3B) and VideoFreeze (Figure 3C) motion indexes sharply increased following the onset of the noise burst (*t* = 0 ms) and remained elevated throughout the duration of the 200 ms noise burst. These responses also coincide with the startle response observed in Figure 3A. In short, the high-speed (Figure 3B) and VideoFreeze (Figure 3C) motion indexes captured the startle response in a similar manner—they both rose sharply and remained elevated during the 200 ms noise burst. The motion index reported by VideoFreeze was relatively larger than that reported by high-speed camera; this is likely because most of the motion that was resolved on a millisecond basis in individual frames in the high-speed recording was captured as motion blur in a single frame in VideoFreeze. Overall, the startle response that was captured using high-speed video was also captured using standard video rates and quantified using the VideoFreeze motion index.

**Figure 4:**
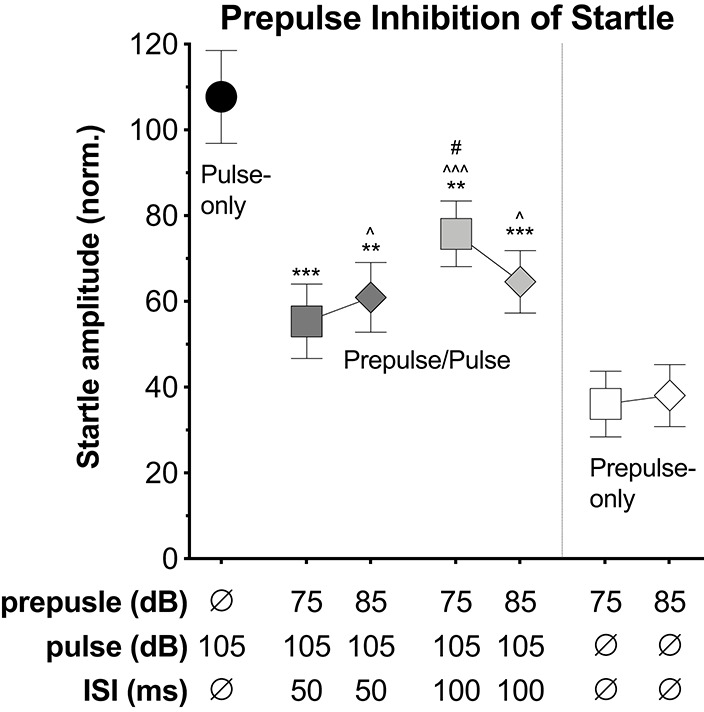
Prepulse inhibition (PPI). Average normalized startle amplitude elicited by the pulse alone, the pulse when preceded by a prepulse, or the prepulse alone. The pulses were 200 ms, 105 dB white noise bursts with a 0 ms rise time. The prepulses were 4 ms, 75 or 85 dB white noise bursts with a 0 ms rise time. The inter-stimulus intervals (ISI) between the prepulse and pulse on prepulse/pulse trials were either 50 or 100 ms. Normalized startle amplitude was significantly lower at all prepulse/pulse conditions relative to the pulse-only condition. Within the two 75 dB prepulse/pulse conditions, normalized startle amplitude was significantly higher at the 100 ms ISI relative to the 50 ms ISI. Normalized startle amplitude was significantly higher at the 75 dB, 100 ms ISI prepulse/pulse condition relative to the 75 dB prepulse-only condition and at both 85 dB prepulse/pulse conditions relative to the 85 dB prepulse-only condition. Starred data points identify significant comparisons against the pulse-only condition (**P < 0.01, ***P < 0.001), the prepulse/pulse condition at the same intensity (dB) (^#^*P* < 0.05), and the prepulse-only condition at the same intensity (dB) (^∧^*P* < 0.05, and ^∧∧∧^*P* < 0.001) using Fisher's LSD.

Lastly, we explored the potential to capture prepulse inhibition (PPI) of the startle response using the VideoFreeze system. Mice were presented with pulse stimuli (200 ms, 105 dB) alone or preceded by a prepulse stimulus (4 ms, 75 or 85 dB, 50 or 100 ms prior to the pulse). In a separate experiment, mice were presented with prepulse stimuli (4 ms, 75 or 85 dB) alone. The pulse and prepulse intensities were selected because the 105 dB noise burst produced significant startle and the 75 and 85 dB noise bursts produced little to no startle during i/o testing (see Figure 1). Figure 4 displays the average normalized startle amplitude elicited by the pulse-only, prepulse/pulse, and prepulse-only stimuli (see Supplementary Data 2 for corresponding data sheet and Supplementary Figure 3 for scatterplot of individual animal data). Normalized startle amplitude differed significantly across stimuli conditions [*F*_(5.12, 814)_ = 8.789, *p* < 0.001]. Compared to the pulse-only condition, the presentation of a prepulse immediately prior to the pulse significantly reduced normalized startle amplitude (*p*-values ≤ 0.007). Within the two 75 dB prepulse/pulse conditions, the 100 ms ISI led to a significantly higher normalized startle amplitude than the 50 ms ISI (*p* = 0.047). There were no significant differences between the other prepulse/pulse conditions (*p*-values ≥ 0.198). Normalized startle amplitude was significantly higher at the 75 dB, 100 ms ISI (but not 50 ms ISI) prepulse/pulse condition relative to the 75 dB prepulse-only condition (*p* < 0.001) and at both 85 dB prepulse/pulse conditions relative to the 85 dB prepulse-only condition (*p*-values ≤ 0.03). Normalized startle amplitude did not differ between the 75 dB, 50 ms ISI prepulse/pulse condition and the 75 dB prepulse-only condition (*p* = 0.124).

## Discussion

Here, we demonstrate the ability to use the VideoFreeze system to elicit and measure the acoustic startle response and PPI of this response in freely moving mice. Mice were first presented with 200 ms white noise bursts of increasing intensities and exhibited no startle responses to lower-intensity stimuli (75 dB to 90 dB) but significant startle responses to higher-intensity stimuli (95 to 120 dB) (Figure 1). We quantified startle amplitude using VideoFreeze's automated assessment of animal movement. Specifically, the maximum motion index during the noise burst was normalized against the maximum motion index immediately prior to the noise burst, which captured rapid yet substantial increases in movement relative to a moving baseline. Similar to previous reports (Valsamis and Schmid, 2011), mice began to startle at 95 dB, and startle amplitude increased with increasing stimulus intensity until reaching a plateau of maximum startle amplitude at 110 dB. The mouse startle response was characterized by significant nose, ear, back, and tail movements that were observed using standard video of 30 Hz and captured quantitively by the normalized startle amplitude (Figure 2, Supplementary Movie 1). In the video still images, the startle response appears as motion blur because the VideoFreeze standard camera temporally integrates all of the motion that occurs over a single frame of 33.33 ms. We believe it is precisely because of this motion blur that VideoFreeze is able to capture and quantify the startle response.

The mouse startle response was also observed using high-speed video of 1,000 Hz, which appeared similar to the response captured by standard video, yet the progression of movement from rostral to caudal was more evident (Figure 3A, Supplementary Movie 2). We compared motion indexes from the high-speed video (Figure 3B) and from VideoFreeze (Figure 3C), and found that despite the differences in video rates, both measures captured the intensity of the mouse startle response observed in Figure 3A. Specifically, the startle response was reflected by a sharp increase in the high-speed and VideoFreeze motion indexes during the startling stimulus. This comparison to high-speed video serves to reinforce that the signal measured in VideoFreeze is in-line with what one would expect based on the higher temporal resolution imaging signal. In addition to observing and quantifying the mouse startle response, we demonstrated the ability to capture prepulse inhibition of acoustic startle response using VideoFreeze. Normalized startle amplitude in response to a strong pulse (200 ms, 105 dB white noise burst) was significantly reduced when the pulse was preceded by a weak prepulse (4 ms, 75 dB or 85 dB white noise burst; 50 ms or 100 ms ISI) (Figure 4).

There can be unexpected variability in motion index scores between individual animals or between trials, however in our experience, a sample size of 16 mice with 1 trial per i/o condition and 10 trials per PPI condition was sufficient for averaging out this variability and detecting startle and PPI (see Supplementary Figures 1, 3 for scatterplots of individual animal data). Future experiments with different parameters (e.g., animal strain, age, size) may introduce more variability and require larger sample sizes and/or more trials.

While this is the first demonstration of using VideoFreeze to quantify the startle in mice, Kirshenbaum et al. (2019) validated the use of VideoFreeze to track and quantify startle and modifications of startle (e.g., PPI and habituation) in zebrafish. Other than this, there are relatively few previous reports of using video to measure the startle response. High-speed video has been used to capture the startle response in various species of fish (Wieland and Eaton, 1983; Hale, 2000; Rice et al., 2011; Chicoli et al., 2014; Hale et al., 2016). High-speed video (Derakhshani and Lovelace, 2010; Bernard et al., 2013) and standard video (Essex et al., 2003; Vousdoukas et al., 2012; Cosić et al., 2016) have also been used in the automated analysis of eye blinks in response to startling stimuli in humans. High-speed video has also been used in conjunction with a piezoelectric startle plate to measure the acoustic startle response in mice (Grimsley et al., 2015), and standard video has been used to detect but not quantify the acoustic startle response in rats (Tovote et al., 2005). Thus, this is the first demonstration of using standard video in the automated assessment of the acoustic startle response in rodents.

The VideoFreeze system is a versatile behavioral testing apparatus that is used extensively to study Pavlovian fear conditioning in rodents (Anagnostaras et al., 2010), and as shown here, may also be a valuable tool for studying startle response. In addition to the capabilities already described, the VideoFreeze system is equipped to present the sound, light, and footshock stimuli required in various startle paradigms (e.g., fear-potentiated startle). Dedicated equipment using stabilimeters still may be more precise than VideoFreeze in assessing startle, and may be a better option for certain experiments such as those requiring high temporal resolution. Nevertheless, we believe that researchers already using the VideoFreeze system will benefit from the additional ability to assess startle in a freely behaving animal without the purchase of new equipment.

## Data Availability Statement

All datasets generated for this study are included in the article/Supplementary Material.

## Ethics Statement

The animal study was reviewed and approved by Institutional Animal Care and Use Committee, University of California San Diego.

## Author Contributions

MP, GH, and SA contributed to the conception and design of the study as well as data interpretation. MP, GH, and KV contributed to data acquisition. MP and GH contributed to data analysis. MP wrote the first draft of the manuscript. All authors contributed to manuscript revision, read, and approved the submitted version.

## Conflict of Interest

Med-Associates Inc. supplied, manufactures and sells the equipment (Video Freeze and related components) described herein. MP, KV, and SA report no conflict of interest and were not funded by Med-Associates Inc. GH is an employee of Med-Associates Inc. Although publication of this paper will indirectly benefit Med-Associates Inc. and its employees, they were not specifically compensated for this activity.

## Supplementary Material

Supplementary Movie 1 The startle response as captured by VideoFreeze.

Supplementary Movie 2 The startle response as captured by a high-speed imaging system.

Supplementary Data 1 Data sheet corresponding to Figure 1 (Input/output function) in the article.

Supplementary Data 2 Data sheet corresponding to Figure 4 (Prepulse inhibition) in the article.

Supplementary Figure 1 Input/output function with individual animal data.

Supplementary Figure 2 Startle amplitude by animal orientation.

Supplementary Figure 3 Prepulse inhibition (PPI) with individual animal data.
